# Ice-Prevention and De-Icing Capacity of Epoxy Resin Filled with Hybrid Carbon-Nanostructured Forms: Self-Heating by Joule Effect

**DOI:** 10.3390/nano11092427

**Published:** 2021-09-17

**Authors:** Catalina Farcas, Oscar Galao, Luigi Vertuccio, Liberata Guadagno, M. Dolores Romero-Sánchez, Iluminada Rodríguez-Pastor, Pedro Garcés

**Affiliations:** 1 Civil Engineering Department, University of Alicante, Ctra. San Vicente s/n, 03690 San Vicente del Raspeig, Spain; catalina.farcas@ua.es (C.F.); oscar.galao@ua.es (O.G.); pedro.garces@ua.es (P.G.); 2 Department of Industrial Engineering, University of Salerno, Via Giovanni Paolo II, 84084 Fisciano, Italy; lvertuccio@unisa.it (L.V.); lguadagno@unisa.it (L.G.); 3 Applynano Solutions, S.L. Parque Científico de Alicante, 03690 San Vicente del Raspeig, Spain; iluminada@applynano.com

**Keywords:** epoxy resin, multi-walled carbon nanotubes, graphite, heating, de-icing, Joule effect

## Abstract

In this study, CNTs and graphite have been incorporated to provide electrical conductivity and self-heating capacity by Joule effect to an epoxy matrix. Additionally, both types of fillers, with different morphology, surface area and aspect ratio, were simultaneously incorporated (hybrid CNTs and graphite addition) into the same epoxy matrix to evaluate the effect of the self-heating capacity of carbon materials-based resins on de-icing and ice-prevention capacity. The self-heating capacity by Joule effect and the thermal conductivity of the differently filled epoxy resin were evaluated for heating applications at room temperature and at low temperatures for de-icing and ice-prevention applications. The results show that the higher aspect ratio of the CNTs determined the higher electrical conductivity of the epoxy resin compared to that of the epoxy resin filled with graphite, but the 2D morphology of graphite produced the higher thermal conductivity of the filled epoxy resin. The presence of graphite enhanced the thermal stability of the filled epoxy resin, helping avoid its deformation produced by the softening of the epoxy resin (the higher the thermal conductivity, the higher the heat dissipation), but did not contribute to the self-heating by Joule effect. On the other hand, the feasibility of electrically conductive epoxy resins for de-icing and ice-prevention applications by Joule effect was demonstrated.

## 1. Introduction

Carbon nanostructured forms such as carbon nanotubes (CNTs) and expanded graphites (EGs) may be used as a “filler” for different materials to confer them different properties, electrical and thermal conductivities and/or mechanical properties among them [1,2,3,4,5,6]. In particular, the dispersion of these kinds of nanoparticles in polymeric systems allows to improve thermal stability, photooxidation resistance and mechanical properties and simultaneously provides the means to make the resulting nanocomposite able to manifest functional properties [7,8,9,10]. Among the different polymeric systems, epoxy resins (which are the most commonly used) belong to polymeric systems which are suited for structural applications [11,12,13,14,15]. These resins have good stiffness, strength, dimensional stability and chemical resistance and durability which makes them useful for a great variety of industrial applications, especially in the electronics, automotive and aerospace industries [16,17]. Applications include protective coatings, adhesives, equipment for the chemical industry, structural composites, electrical laminates and electronic packaging [18,19,20].

However, as polymers, epoxy resins are insulating materials, which limits their use for some applications [21]. In recent years, nanoparticles, nanotubes or nanofibers have been considered as additives or fillers for epoxy matrices to produce high-performance composites with enhanced mechanical properties [22,23,24] and advanced and multifunctional properties such as electrical conductivity and self-responsiveness capacity [25,26], which are interesting for innovative industrial applications [27]. Some other key properties of epoxy resins containing carbon-based nanoparticles are the increased adhesion properties [28], heat dissipation capacity due to the increased thermal conductivity [29,30], higher durability (higher thermal stability), antibacterial properties [31] and fire and corrosion resistances [32,33].

A suitable amount of unfunctionalized or functionalized CNTs or expanded graphite with controlled values of aspect ratio is able to confer to the hosting epoxy material many self-responsive functions such as self-protection, self-sensing [15,34], self-healing [8], anti- or de-icing [10,35], etc.

It is expected that a hybrid combination of these two kinds of fillers allows obtaining synergic effects from many points of view, such as a lower electrical percolation threshold (EPT), maintaining good thermal and electrical conductivity or de-icing performance. In fact, it has been found that an 8:2 weight ratio mix of CNTs and graphene-based nanoplatelets in epoxy resin determines a synergistic enhancement of electrical percolation and conductivity, manifesting better properties than the 10:0 and 0:10 weight ratios [36]. This strategy based on the possibility of maximizing specific performance through synergic effects appears to be promising for many researchers [4,37,38,39,40].

Recently, Prolongo et al. and Redondo et al. proposed the doping of epoxy resins used as coatings with graphene nanoplatelets (GNP, 8–10 wt.%) or CNTs (0.1–0.5 wt.%) for the self-heating of epoxy resin by Joule effect. They demonstrate the higher effectiveness for de-icing and anti-icing applications of CNTs due to their higher electrical conductivity, reaching higher temperatures at lower electrical voltages, compared to the effect produced by GNPs which, differently, shows lower but more homogeneous heating [41,42]. This is explained due to the higher thermal conductivity of GNPs compared to that of CNTs.

The influence of CNTs or graphite on the self-heating capacity of an epoxy resin incorporating one of the fillers has been investigated by Vertuccio et al. [10]. They conclude that CNTs are the best performing materials to provide epoxy resin with self-heating properties (for similar electrical conductivity values, lower dosage of CNTs required). On the contrary, despite the higher dosage required for graphite, this material generates lower viscosity for the epoxy resin compared to that of CNTs (lower dosage).

Scientific literature search indicates that there are very few publications related to the simultaneous incorporation of different types of carbon-based nanomaterials in epoxy resins [43]. The majority of these papers investigate the synergistic effects of CNTs and GNPs in mechanical properties [36,44], concluding the benefits in terms of flexural and electrical properties of the simultaneous incorporation of both types of fillers. However, they do not analyze the effect of different filler incorporation on the self-heating capacity of epoxy resins.

Other authors [45,46,47] analyze the effect of CNTs and GNPs on the thermal properties of epoxy resins, obtaining increased thermal conductivity values for the hybrid composites, which is explained by the increased surface area between CNT, GNPs and the epoxy matrix, avoiding the re-stacking and agglomeration of GNPs.

The approach in this work is to evaluate the influence of incorporating two different types of carbon-based materials, CNTs and expanded graphite, in the same polymer matrix, an epoxy resin, simultaneously considering the high electrical conductivity of CNTs and the high thermal conductivity of graphite. Fillers with different morphology and aspect ratio provide the insulating epoxy resin with electrical properties and consequently, self-heating capacity by Joule effect (heat release of a material due to the movement of electrons when applying an electrical voltage, which is a derived property of electrical conductivity) for de-icing and ice-prevention capacity. Moreover, geometry, size and aspect ratio of CNTs and graphite were also considered as key parameters in the analysis of the self-heating properties of the corresponding epoxy resins.

## 2. Materials and Methods

### 2.1. Materials

A commercial two-component epoxy resin (Prime^TM^ 180, Gurit (UK) Ltd., Newport, UK) was used as the thermosetting matrix for the incorporation of CNTs and/or graphite. Table 1 includes the main properties for the unmixed epoxy resin and the corresponding hardener (data from provider). Multiwall carbon nanotubes (Graphistrength C100, provided by Arkema, Table 2) and purified expanded graphite (ABG1010, Superior Graphite, IL, USA, Table 3) were selected and independently and simultaneously incorporated in different proportions into the epoxy resin. Data from providers indicate an average aspect ratio of 500 and 55, respectively, for C100 and ABG1010. A high shear process was used for mixing the particles with the epoxy resin. Finally, the curing agent (hardener) was added in the ratio recommended by the provider (Table 1) to the differently filled epoxy resins. The curing process was carried out in two steps, a pre-cure cycle of 20 min at 120 °C to generate enough green strength for de-molding, followed by a post-cure of 180 °C for 1 h to maximize the thermal properties of the system.

### 2.2. Experimental Procedure

A high-shear dispersion process was used for the incorporation of CNTs and graphite independently or simultaneously into the epoxy resin until homogeneous dispersion (5000 rpm, 30 min). Different particle concentrations (wt.%) and ratios were used, as indicated in Table 4. Once the different epoxy resin dispersions containing CNTs and/or graphite were prepared, the curing agent was added (weight ratio 100:100), and the differently filled epoxy resins were cast for curing in corresponding shape and dimension molds for characterization with the different experimental techniques. Disk-type specimens were prepared to measure the electrical conductivity of mixtures with only CNT or graphite, separately. Parallelepipedal-type specimens were prepared for the heating tests, which included the measurement of electrical resistivity.

### 2.3. Characterization

For electrical characterization, the first approach was performed by measuring the electrical conductivity of different concentrations of CNT or graphite in the epoxy resin matrix, separately. After that, both carbon products were mixed in different proportions into the epoxy resin matrix, and electrical conductivity (or its inverse, electrical resistivity) and thermal conductivity were measured. In all cases, DC was used.

As for the aforementioned first approach, the measurements were performed on disk-shaped specimens of about 2 mm of thickness and 50 mm of diameter. In order to reduce the possible effects due to surface roughness and to ensure ohmic contact with the measuring electrodes, the specimens were coated by using a silver paint with a thickness of about 50 μm and characterized by surface resistivity of 0.001 Ω·cm. The measurement apparatus for DC characterization of specimens above the percolation threshold was composed of a Keithley 6517 A multimeter with the function of a voltage generator (max ±1000 V) and voltmeter (max ±200 V) and an HP34401 A ammeter (min current 0.1 µA). Below the percolation threshold, the system was composed exclusively of a Keithley 6517 A multimeter working as a voltage generator and pico-ammeter (min current 0.1 fA).

In this work, electrical characterization was performed to obtain the range of nanofiller concentrations suitable to impart different functions to the materials. The well-known percolation theory can be applied to explain the electrically conducting behavior of composites consisting of conducting fillers and insulating matrices. The concentration of the conducting filler must be above the percolation threshold in order to achieve conducting networks in the composite.

Electrical and thermal conductivities of the differently filled epoxy resins were measured after curing to evaluate the influence of the incorporation of different concentrations of CNTs and the simultaneous presence of graphite. Secondly, ice-prevention and de-icing tests were carried out to evaluate the self-heating capacity of the filled epoxy resins.

Electrical resistivity of the specimens tested for heating, prevention and de-icing was measured by the 4-point method. A digital multimeter and a voltage source were used for measurements, with copper clips being applied to the silver conductive painted epoxy surface. Electrical resistivity (Ω·cm) of the specimens was calculated for the range of 30–300 V. For the evaluation of Joule effect (self-heating capacity), the temperature on the surface of each epoxy resin was measured continuously (until 30 min) during the application of different voltages.

Thermal conductivity was evaluated by using a C-Therm TCi device (Mathis Instruments Ltd.) with a universal sensor for liquid and solid specimens.

### 2.4. Heating Test

Heating tests consisted of applying a fixed voltage through the outer electrodes while continuously monitoring the current and the temperature in different parts of the specimen and the room.

The heating tests were carried out at room temperature. For this research, four specimens were manufactured for each batch. The specimens were previously weighed (40.18 ± 3.48 g) and measured (13 cm × 3 cm × 1 cm). The sides (3 × 1 cm^2^) were painted with silver paint in order to improve electrical contact (0.5 mm thick copper plate and 2 mm thick carbon felt). To perform the heating tests, different constant voltages in direct current (DC) were applied between the two ends of the conductive epoxy resin (3 × 1 cm^2^).

The neat epoxy resin was not considered in this study, as its resistivity is higher than 1·10^11^ Ω·cm, indicating a lack of electrical conductivity and suitability for heating applications by Joule effect.

The temperature of the specimens was continuously monitored by four resistance temperature detectors (RTD) type Pt100 which were connected to a data logger (model DAS-8000, Design Instruments, Barcelona). Two resistance temperature detectors (RTD) were placed at the center of the upper and lower sides of the specimen, and the other two near the ends of the upper side. Additionally, the environmental temperature was registered by two other RTDs. A thermographic camera (model Flir E30) was used to check the uniformity of heat distribution.

Different constant voltages were applied to the specimens with a digital direct power source. The electrical current was monitored with Keithley 2002 digital multimeters throughout the test.

In all cases, four different specimens of each composite were tested, and the tests were repeated at least three times.

### 2.5. Ice-Prevention and De-Icing Tests

After the heating tests, ice-prevention tests were carried out. Ice-prevention tests consisted of applying a fixed DC voltage in order to keep the specimen’s temperature above +3 °C to avoid the formation of ice. A Liebherr freezer with internal dimensions of 1.45 m × 0.50 m × 0.65 m was used to carry out the ice-prevention and de-icing tests. The freezer’s average environment temperature was −15 °C. First, the specimen, at environmental temperature (i.e., 20 °C, approximately) was introduced into the freezer. When it reached +5 °C, the power source was turned on at a fixed voltage. Once a stable temperature was reached, the power supply was turned off. The evolution of the temperature was recorded continuously during each test.

After ice-prevention tests, de-icing tests were performed. The specimens were kept in the freezer for a period of approximately 24 h, reaching an initial temperature of −15 °C. Then, the direct power source was turned on at a fixed voltage. Like in the prevention tests, the aim was to keep the specimen’s temperature above +3 °C to avoid the formation of ice. After five hours, the power was turned off.

During all tests, the temperature and the electrical current were constantly monitored. All test types were performed with DC.

## 3. Results

### 3.1. Study of the Electrical Conductivity of the Epoxy Resin with CNT and Graphite Separately

Electrical resistance was calculated from the evaluation of the slope of the linear regression fit from the experimental data of the voltage-current measurements for all considered systems of epoxy resin containing different dosages of CNT and graphite (specimens EC1 to EC11, Table 4).

**Figure 1:**
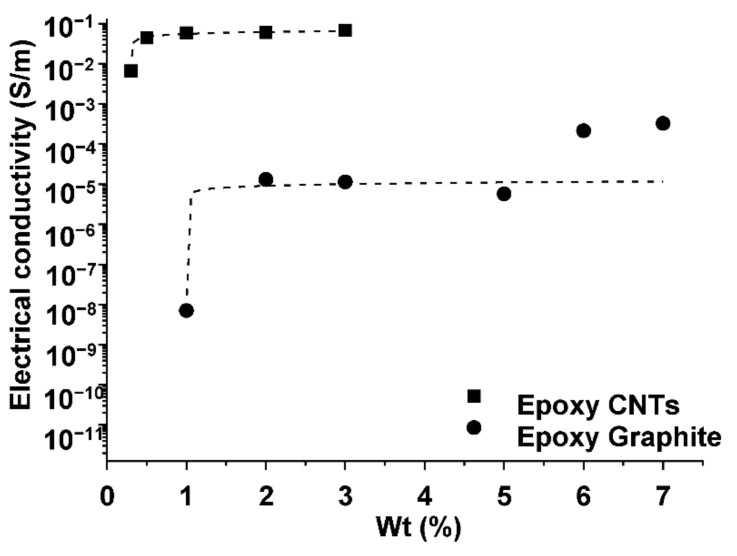
Electrical conductivity trend of the CNTs system and graphite system.

The results of the average electrical conductivity are shown in Figure 1. The volume electrical conductivity (*σ*) was calculated using the evaluated electrical resistance (*R*) and the specimen dimensions as:

$$\sigma =\frac{L}{A*R}$$

where *A* is the cross-sectional area of the specimen and *L* is the specimen’s thickness.

In Figure 1, the electrical conductivity of the epoxy resin (*σ*) as a function of the amount (wt.%) of fillers (CNTs or graphite) is reported. As expected by the percolation theory, the conductivity depended on the filler loading, in agreement with a scaling law mentioned above. Values of about 3.3 × 10^−2^ and 3.2 × 10^−4^ S/m were achieved at the highest filler loading for the CNT system (i.e., 3 wt.%) and the graphite system (i.e., 7 wt.%), respectively. The systems exhibited the typical abrupt increase of conductivity predicted by the percolation theory, with an electrical percolation threshold of x_c_ < 0.5% by weight for the CNT system and x_c_ < 2.0% by weight for the graphite system. The results shown in Figure 1 indicate the range suitable to impart a self-heating function to the epoxy resin, in particular, a concentration of filler higher than 0.5 wt.% for the CNTs system and a concentration of filler higher than 2 wt.% for the graphite system.

### 3.2. Study of the Heating Function in Epoxy Resin

According to the concentrations obtained in the experiment below for the electrical resistivity of epoxy resin incorporating CNTs or graphite separately, concentrations from 0.25 to 1 wt.% of CNTs and/or 5 wt.% of graphite were considered for the self-heating and de-icing experiments.

The self-heating capacity of the specimens due to electrical conductivity by Joule effect was analyzed.

A summary of the electrical characteristics (resistivity, current type and fixed voltage applied), temperature variation and energy characteristics (average power, consumed energy and average energy consumption) of the heating tests of specimens RP1, RP2, RP3 and RP4 is included in Table 5.

The first part of the study was the evaluation of the electrical resistivity of the different specimens. The results are included in Table 5, where lower values of electrical resistivity for the specimens containing a higher percentage of C100 (1% CNTs, i.e., RP1 and RP4) can be observed. However, compared to RP4 (1% C100 + 5% ABG1010), the quantum leap in the electrical resistivity of RP2 specimens (0.25% C100) and RP3 specimens (0.5% C100) indicates that these concentrations were below the percolation threshold, as previously observed in Figure 1. The presence of graphite did not seem to have a great effect on the electrical resistivity of the specimens. RP4 (1% C100 + 5% ABG1010) showed only a slight decrease in electrical resistivity compared to that of RP1 (1% C100). Therefore, the electrical conductivity of the epoxy resin matrix could be mostly assigned to CNTs (Table 5).

The tests were carried out by applying different fixed DC voltages (50, 70 and 100 V) while monitoring the electrical current, surface temperature of the specimens and the environmental temperature. As expected, the results showed that the higher the voltage applied to the specimens, the higher the temperature increase.

**Figure 2:**
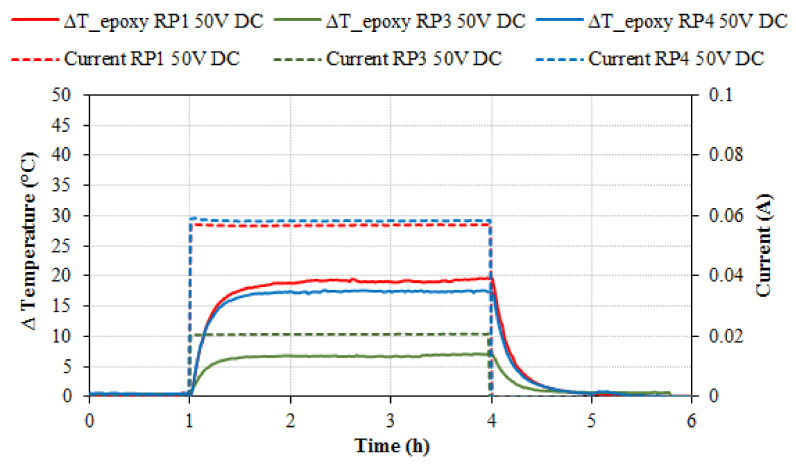
Specimen’s temperature increment (°C) and electrical current (A) versus time (h) for 50 V DC heating tests with RP1, RP3 and RP4 specimens.

Figure 2 shows different heating tests carried out by applying 50 V DC with specimens RP1, RP3 and RP4. Tests at 50 V DC with RP2 series showed negligible temperature increments. The RP1 specimens registered an increment of +5 °C in 4 min, and the maximum temperature (an increment of +19.7 °C) was observed in less than one hour, with a steady level of electrical current (0.057 A). The RP4 specimens showed similar results: the increment of +5 °C was reached in 4 min, and the maximum temperature was +17.6 °C with a steady electrical current of 0.058 A. The increment of +5 °C of the RP3 specimens was registered after 12 min with an electrical current 0.021 A (the maximum increment was +7.1 °C) (Table 5). All the specimens reached the maximum temperature in less than one hour. The electric current and the voltage were kept constant throughout the tests, hence it can be held that the electrical resistivity of the composites remained constant in the tested temperature range.

**Figure 3:**
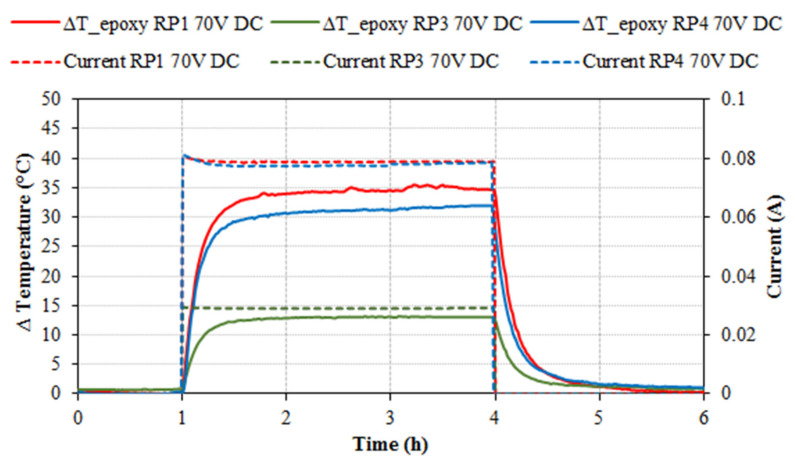
Specimen’s temperature increment (°C) and electrical current (A) versus time (h) for 70 V DC heating tests with RP1, RP3 and RP4 specimens.

The specimens were also tested by applying 70 V DC, as shown in Figure 3. Once more, the RP2 series showed negligible temperature increments with this voltage. It can be observed that the specimens RP1 and RP4 presented similar results, with temperature increments of +35.5 and +31.9 °C and with electrical currents of 0.079 and 0.078 A, respectively. The specimens RP3 with 70 V DC presented an increment of +13.1 °C with an electrical current of 0.029 A (Table 5). In cold regions with environment temperatures between −5 and −10 °C, increasing the temperature of the epoxy resin by approximately +15 °C might be enough to prevent ice formation.

**Figure 4:**
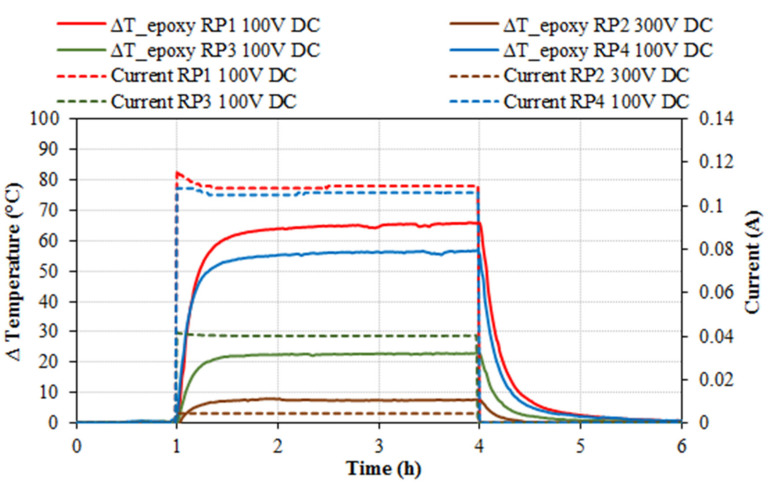
Specimen’s temperature increment (°C) and electrical current (A) versus time (h) for 100 V DC tests for epoxy resin RP1, RP3 and RP4, and 300 V DC for epoxy resin RP2 (specimens’ size 13 × 3 × 1 cm).

Results of the heating tests by applying 100 V DC are shown in Figure 4. RP2 series showed negligible results, but for comparison, RP2 tests at 300 V DC are included in Figure 4. In this case, despite the high applied voltage, the increase in temperatures was very small, hence the RP2 series was not tested for prevention and de-icing applications.

It can be observed that RP1 showed the highest temperature increment (+66.0 °C for RP1, +56.8 °C for RP4 and +23.2 °C for RP3). The temperature increment was faster (temperature gain velocity) for RP4. Taking +20.0 °C as reference, this increment was reached in 3 min for RP4, in 4 min for RP1 and in 16 min for RP3. The electrical current monitored throughout the tests was steady, with average values of 0.106 A for RP4, 0.109 A for RP1 and 0.040 A for RP3 (Table 5). All the specimens reached the maximum temperature in less than one hour.

It can be observed that for the three applied voltages (50, 70 and 100 V), the trend in the temperature increase was the same. RP1 specimens were the ones with the higher temperature increase, RP2 specimens had negligible heating and RP3 specimens had intermediate heating. This trend completely agrees with the electrical conductivity values obtained for the specimens. The higher the electrical conductivity (the lower electrical resistivity), the higher the self-heating capacity. However, for the three studied voltages, the RP4 specimen, with content of CNTs (1% C100) and electrical resistivity values similar to those of RP1 (Table 5), showed a lower temperature increase than RP1 specimens did. This result indicates that the presence of graphite in the formulation of RP4 specimens seemed not to affect the electrical conductivity of the epoxy resin, but the heating effect.

**Figure 5:**
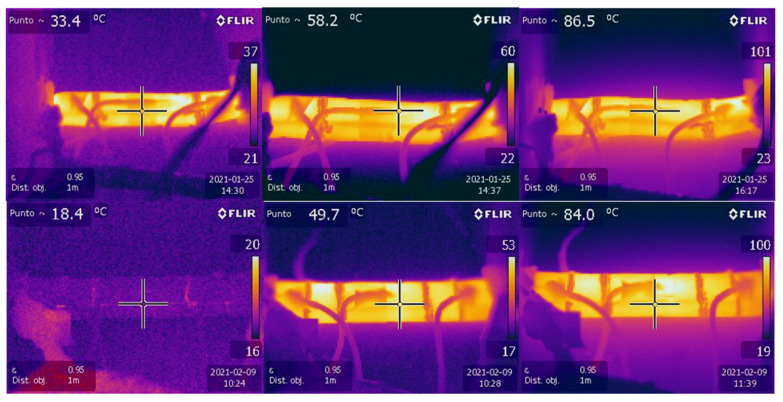
Thermographic pictures of different tests with different specimens (RP1 upper, RP4 lower).

As indicated, the tests were also monitored with an infrared camera. In Figure 5, the consistent increasing temperature with time can be observed from thermographic pictures taken during the heating tests. The upper pictures were taken at different times during a RP1 specimen heating test, in which a fixed voltage of 100 V DC was applied. The lower pictures were taken at different times during a RP4 specimen heating test, in which a fixed voltage of 100 V DC was applied. In both cases, as expected, an increment in temperature could be observed with time, in degrees Celsius. It is worth noticing that specimen RP1 at 86.5 °C showed severe deformation (upper right picture). For that reason, RP1 specimens were discarded for ice-prevention and de-icing tests, even though they reached higher temperature increments than RP3 did.

**Figure 6:**
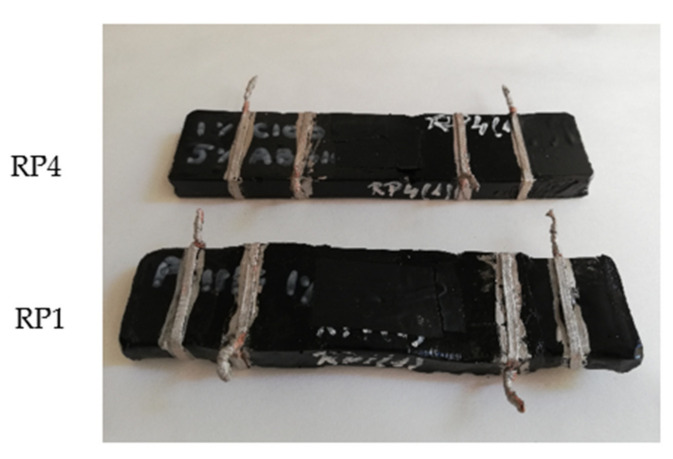
Image of an RP1 (1% CNT addition) specimen (lower) and an RP4 (hybrid 1% CNT + 5% graphite addition) specimen (upper) after heating tests.

Figure 6 shows a picture of the former RP1 and RP4 specimens, with their four silver-paint-copper electrodes, after heating tests. As can be observed, and was previously commented, RP1 (1% C100) was seriously deformed after the 100 V DC heating tests (maximum temperature of 94 °C was reached). The shape of the RP4 specimen (1% C100 + 5% ABG1010) remained stable even though the maximum reached temperature was similar (90 °C).

The different aspect and heating response of RP1 and RP4 specimens may be explained by the presence of graphite in the formulation of RP4 (1% C100 + 5% ABG1010) compared to the formulation containing only CNTs as additive in RP1 specimens (1% C100). In addition to the measurements of electrical conductivity (resistivity), thermal conductivity of the four specimens was evaluated. The results are included in Table 6. Thermal conductivity of the RP1 specimen (1% C100) showed a lower value (0.224 W/m/K) compared to those of the RP2, RP3 and RP4 specimens, which showed higher thermal conductivity values due to the presence of graphite and, to a much lesser extent, the increased percentage of CNTs. Comparison between RP1 and RP4 specimens indicated that the faster heat dissipation in the RP4 specimen due to its higher thermal conductivity provided by the graphite helped avoid its deformation because of the heating effect produced by electrical resistivity.

**Figure 7:**
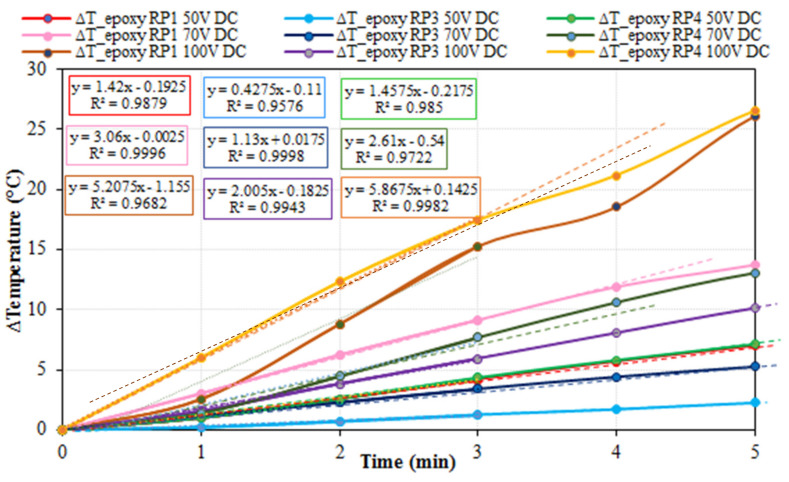
Specimens’ average incremental temperature (for RP1, RP3 and RP4 specimens) and electrical current versus time for 50 V, 70 V and 100 V fixed voltage DC tests. The dotted lines represent the linear regression function for the first 3 min of data. Equations and coefficients of determination (R^2^) are also included in the dotted lines with corresponding colors.

Another important aspect in heating processes is heating velocity (temperature increase over time), which was calculated for all the heating tests. The first five minutes of the formerly shown heating tests (RP1, RP3 and RP4 at 50, 70 and 100 V DC) are depicted in Figure 7, where regression lines, formulas and R^2^ coefficients are included. As it can be observed, RP1 and RP4 showed similar heating velocities for each maximum voltage applied, whereas RP3 showed much lower results. Hence, with the studied conditions, the thermal conductivity did not seem to play an important role in heating velocity. Furthermore, given the fact that RP3 (with half the amount of CNTs) heated up slower, apparently, it is electrical conductivity that dominates the Joule effect.

**Figure 8:**
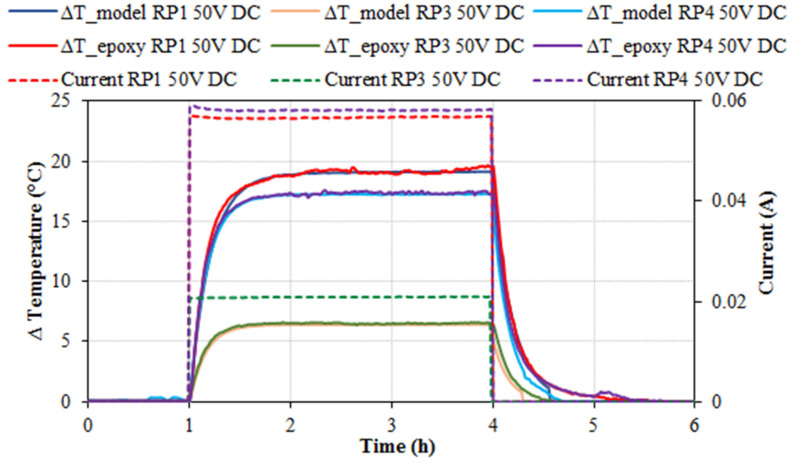
Specimen’s and model’s temperature increment (°C) and electrical current (A) versus time (h) for 50 V DC heating tests with RP1, RP3 and RP4 specimens.

In previous studies with a different matrix, a mathematical model which predicts that the degree of heating is adjustable with the applied voltage by means of classical physics equations (Fourier, Newton and Joule) was applied to simulate the heating behavior [48,49]. Using the equations of this model, all tests were simulated in order to verify that it could be used in different materials. Figure 8 overlays the results of Figure 2 (ΔT and electrical current vs time at 50 V DC for RP1, RP3 and RP4 series) and the results obtained using the aforementioned mathematical model. It can be observed that the model perfectly matched the experimental results at the three classical stages (heating, steady-state and cooling), which leads to the conclusion that the aforementioned model could be used with the composites studied in the present research. For instance, the optimized conductive epoxy resin material could be determined with this tool by analyzing the minimum power required to obtain the desired temperature.

### 3.3. Study of Ice-Prevention and De-Icing for Epoxy Resin RP4 (1% C100 + 5% ABG1010) and RP3 (0.5% C100 + 5% ABG1010)

**Figure 9:**
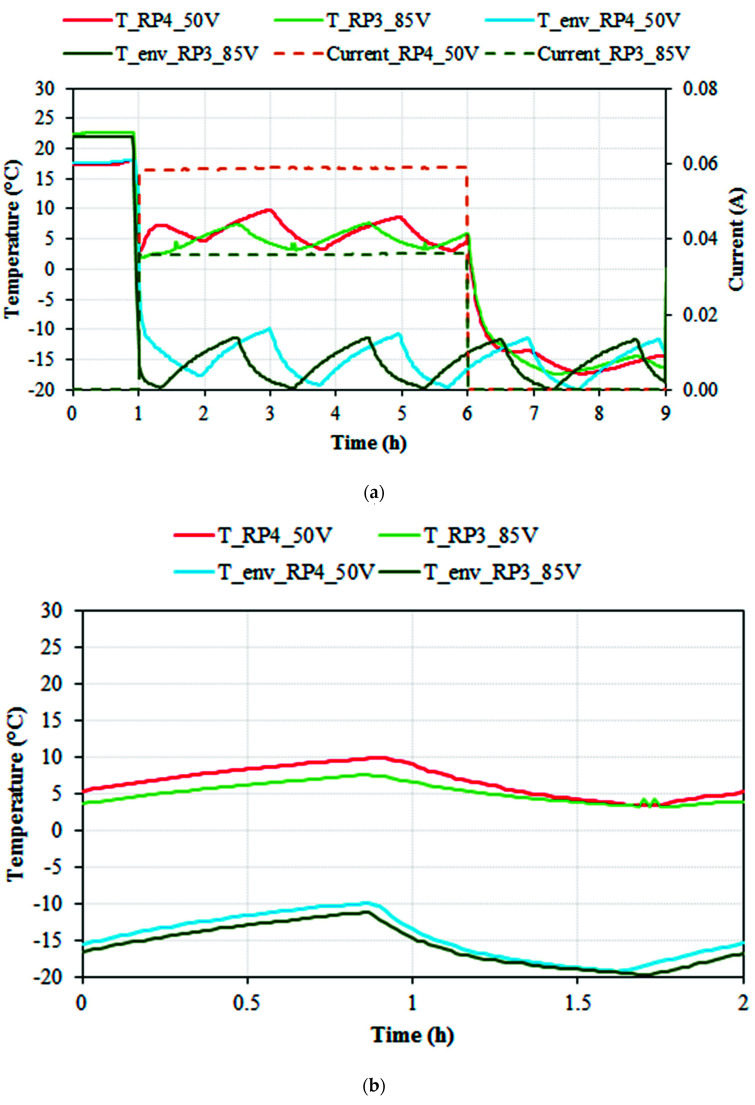
Ice-prevention test. (**a**) Environmental (freezer) temperature (T_env_RP4_50 V and T_env_RP3_85 V) in °C, specimen’s temperature (T_RP4, T_RP3) in °C, and monitored current in A versus time in hours, for RP4 at 50 V DC and RP3 at 85 V DC ice-preventing tests. (**b**) Detail of one single cycle for each test, focused, scaled and moved to zero-time origin.

Figure 9a shows two ice-prevention tests with RP3 and RP4 epoxy resin specimens. Specimens and environmental temperature (in °C) and electrical current (in A) are denoted versus time (in hours). The voltages shown (50 V DC for RP4 and 85 V DC for RP3) were the minimum to increase the specimen’s temperature above +3 °C in order to prevent the formation of ice, indicating that the RP4 specimen with the higher percentage of CNTs (1% CNTs) compared to that of RP3 (0.5% CNTs) required a lower voltage for heating and avoiding the formation of ice.

The maximum temperature registered for RP4 was 9.8 °C and 7.6 °C for RP3, with an average current of 0.0588 and 0.0359 A, respectively. The calculated average power was similar for RP4 and RP3 specimens (250 and 250 W/m^2^, respectively). The periodic tendency observed in the environmental temperature was due to freezer regulation hysteresis.

Since it is difficult to compare both shown tests due to the hysteresis of the freezer, a detail of one single cycle for each test was focused, scaled and moved to zero-time origin, as shown in Figure 9b. It can be observed that the behavior of RP3 and RP4 specimens was very similar. Nonetheless, the fact that the RP3 specimen required higher voltage and lower electrical current to obtain similar results is worth noting.

**Figure 10:**
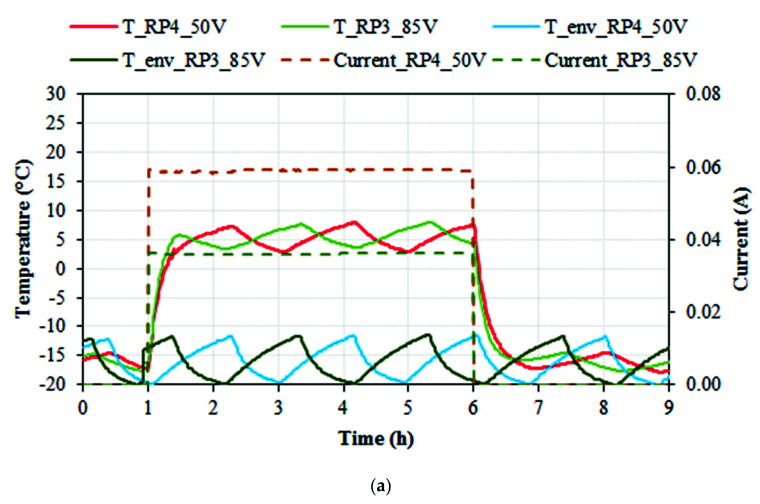

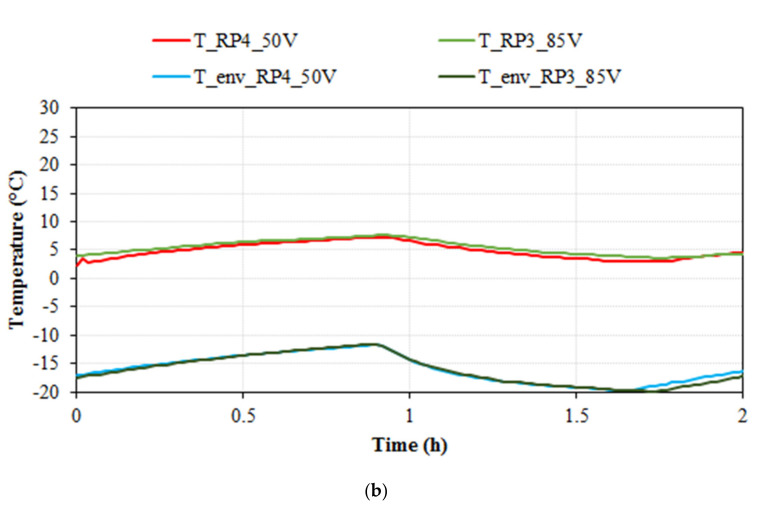
De-icing test. (**a**) Environmental (freezer) temperature (T_env_RP4_50 V and T_env_RP3_85 V) in °C, specimen’s temperature (T_RP4, T_RP3) in °C, and monitored current in A versus time in hours, for RP4 at 50 V DC and RP3 at 85 V DC de-icing tests. (**b**) Detail of one single cycle for each test, focused, scaled and moved to zero-time origin.

Experimental conditions and results obtained for the de-icing tests are represented in Figure 10. Specimens and environmental temperature (in °C) and electrical current (in A) are denoted versus time (in hours). The voltages shown (50 V DC for RP4 and 85 V DC for RP3) were the minimum to increase the specimen’s temperature above +3 °C. As expected, voltages and temperature were the same as the required in the ice-preventing tests, considering that the system temperature (environment) was the same. As it can be observed, when applying a 50 DC fixed voltage, RP4 specimens were able to increase their temperature above 0 °C in 28 min, and when applying an 85 DC fixed voltage, RP3 specimens were able to increase their temperature above 0 °C in 24 min. Temperatures above +5 °C were obtained in 53 min and 34 min for RP4 and RP3 specimens, respectively, but freezer hysteresis should be taken into consideration. For RP4 specimens, the average temperature was 5.4 °C, and the maximum temperature registered was 7.9 °C, whereas for RP3 specimens, the average temperature was 5.5 °C, and the maximum temperature registered was 8 °C, i.e., almost identical. The average current for RP4 was 0.0590 A and 0.0360 A for RP3, with an average power of 251 W/m^2^ for RP4 and 253 W/m^2^ for RP3. After five hours the electrical power source was turned off. This test type was performed with different specimens of each resin system, obtaining negligible differences in behavior among the samples, indicating the reproducibility of the test and results. These results are very similar to those obtained in ice-prevention tests. The periodic tendency observed in the environment temperature was due to freezer regulation hysteresis. Similar to Figure 9b, Figure 10b shows a detail of one single cycle of the de-icing tests for the RP4 specimen with 50V and the RP3 specimen with 85 V. It can be observed that the specimens presented stable and almost identical behavior throughout the tests.

Table 7 contains the results for ice-prevention and de-icing tests, including the average consumption of energy, the consumed energy, the average power, the average absolute temperature and average temperature increment reached during the tests, the fixed voltage applied and the resistivity of the specimens.

Considering a cost value of 0.15 EUR/kW·h the average cost for RP4 would be 0.1878 EUR/m^2^ and 0.1892 EUR/m^2^for RP3. The consumed energy and the temperatures reached were also similar for the two sets of specimens with different voltages, 85 V for RP3 (0.036 A) and 50 V for RP4 (0.059 A) (Table 7). This indicates that the higher percentage of CNTs in RP4 specimens compared to those in the RP3 specimens allowed higher electrical conductivity and therefore lower energy consumption to achieve the same effects for de-icing and ice-prevention tests.

As it can be observed from Table 7, RP3 and RP4 specimens showed very similar results regarding temperature and energy, but with different electrical parameters. As expected, higher electrical resistivity implies a need of higher voltages (and lower electrical currents, as can be observed in Table 5), to obtained pre-set results. Depending on the surface to cover, the former could modify the devices needed to apply the required power to the system. Moreover, RP3 contains half the amount of CNTs in RP4, which provides an important economic saving.

## 4. Conclusions

1. RP3 specimens, with the hybrid addition of 0.5% C100 + 5% ABG1010, and RP4 specimens, with the hybrid addition of 1% C100 + 5% ABG1010, significantly increased their temperature by Joule effect with relatively low DC voltages and electrical currents. Although both composites would be feasible for heating applications, the self-heating capacity was more noticeable in the RP4 specimen, with the highest value of electrical conductivity. The highest percentage of CNTs in the formulation of the RP4 specimen compared to that of the RP3 specimen led to the highest self-heating effect.
2. RP4 specimens with the same electrical conductivity as RP1 specimens (same CNTs concentration, 1% C100) did not deform at high temperatures (higher than 80 °C) due to the softening of the epoxy resin thanks to the presence of graphite with high thermal conductivity, allowing heat dissipation. RP1 specimens showed severe deformation due to the absence of graphite (no heat dissipation was produced).
3. Results have shown that the RP3 and RP4 specimens were able to maintain their temperature above 0 °C in an environmental temperature of −15 °C if a suitable voltage was applied. Therefore, these composites would be feasible for ice-prevention and de-icing applications with a relatively energy low cost (for five hours of testing, the average price was calculated to be 0.94 EUR/m^2^ for both RP3 and RP4).
4. Specimens with the same amount of CNTs showed similar electrical conductivity and similar temperature and energy behavior regardless of the quantity of graphite, indicating that CNTs were the carbon-based material responsible for the electrical conductivity and, consequently, the self-heating capacity by Joule effect.
5. It is feasible to model the self-heating of carbon-based epoxy resins in order to validate the mechanism of heating. An increment of +17 °C was obtained for RP4, with a fixed voltage of 50 V.

## Figures and Tables

**Table 1:** Main properties of the unmixed epoxy resin Prime^TM^ 180 and the corresponding hardener.

Property	Epoxy Resin	Hardener
Mix ratio (parts by weight)	100	100
Initial viscosity at 25 °C (cP)	617	80
Density (g/cm^3^)	1.16	1.19
Mixed density (g/cm^3^)	1.17	

**Table 2:** Main properties of the carbon nanotubes incorporated into the epoxy resin.

Property	Value
Outer diameter (nm)	10–15
Mean length (mm)	1
Mean nº of walls	5–15
C content (%)	>90
Apparent density (kg/m^3^)	50–150
Mean agglomerate size (mm)	200–500
Aspect ratio	>>>100
Fe content (%)	4–7

**Table 3:** Main properties of the graphite incorporated into the epoxy resin.

Property	Value
Loss of ignition (%)	99.9
Ash (%)	0.01
Moisture (%)	<0.1
True density (g/cm^3^)	2.25
Scott volume (g/cm^3^)	0.043
Surface area (m^2^/g)	22
Sulfur (%)	0.1
Size distribution (µm)	D10 3.3
	D50 9.8
	D90 40.2

**Table 4:** Concentration of CNTs and graphite independently and simultaneously incorporated into the epoxy resin for each specimen type.

**Nomenclature**	**wt.% CNTs**	**wt.% Graphite**	**Specimen Type**
RP1	1	-	Parallelepipedal
RP2	0.25	5	Parallelepipedal
RP3	0.5	5	Parallelepipedal
RP4	1	5	Parallelepipedal
EC1	0.3		Disk
EC2	0.5		Disk
EC3	1		Disk
EC4	2		Disk
EC5	3		Disk
EC6		1	Disk
EC7		2	Disk
EC8		3	Disk
EC9		5	Disk
EC10		6	Disk
EC11		7	Disk

**Table 5:** Summary of the electrical characteristics (resistivity, current type and fixed voltage applied), temperature variation and energy characteristics (average power, consumed energy and average energy consumption) for the heating tests. The calculated test cost in EUR is also included considering a value of 0.15 EUR/kW·h.

Heating								
Type	Resistivity (Ω·cm)	Voltage (V) DC	Current (A)	ΔT (°C)	Average Power (W/m^2^)	Consumed Energy (kW·h)	Cost (EUR)	Average Energy Consumption (kW·h/m^2^)
RP1 1%C100	192	50	0.057	19.7	247.1	8.46·10^−3^	1.27·10^−3^	0.737
	194	70	0.079	35.5	480.0	16.4·10^−3^	2.47·10^−3^	1.432
	201	100	0.109	66.0	948.5	32.5·10^−3^	4.87·10^−3^	2.830
RP2 0.25% C100 + 5%ABG1010	14231	250	0.004	6.5	78.1	2.8·10^−3^	0.42·10^−3^	0.233
	14147	300	0.005	7.9	113.2	4.0·10^−3^	0.60·10^−3^	0.338
RP3 0.5% C100 + 5%ABG1010	510	50	0.021	7.1	85.7	3.1·10^−3^	0.46·10^−3^	0.254
	515	70	0.029	13.1	166.5	5.2·10^−3^	0.78·10^−3^	0.427
	525	100	0.040	23.2	333.0	12.0·10^−3^	1.80·10^−3^	0.988
RP4 1% C100 + 5%ABG1010	184	50	0.058	17.6	247.6	8.7·10^−3^	1.30·10^−3^	0.739
	184	70	0.078	31.9	462.8	16.1·10^−3^	2.42·10^−3^	1.373
	184	100	0.106	56.8	902.4	31.7·10^−3^	4.75·10^−3^	2.692

**Table 6:** Thermal conductivity and electrical resistivity of the RP specimens.

Type	Electrical Resistivity (Ω·cm) at 50 V (Excepting RP2 at 300 V)	Thermal Conductivity (W/m·K)
RP1 1%C100	192	0.224
RP2 0.25% C100 + 5% ABG1010	14,147	0.351
RP3 0.5% C100 + 5% ABG1010	510	0.370
RP4 1% C100 + 5% ABG1010	184	0.391

**Table 7:** Summary of the electrical (resistivity and fixed voltage applied), temperature (variation and absolute) and energy characteristics (average power, consumed energy and average energy consumption) of ice-prevention and de-icing tests.

	Type	Resistivity (Ω·cm)	Voltage (V) DC	ΔT (°C)	T (°C)	Average Power (W/m^2^)	Consumed Energy (kW·h)	Average Consumption (kW·h/m^2^)
Ice-prevention	RP3 0.5% C100 + 5% ABG1010	501	85	21	4.8	252	1.53·10^−2^	1.260
De-icing		497	85	21.4	5.5	253	1.53·10^−2^	1.263
Ice-prevention	RP4 1% C100 + 5% ABG1010	182	50	21.2	6.2	250	1.47·10^−2^	1.249
De-icing		181	50	21.3	5.4	251	1.48·10^−2^	1.255

## Data Availability

The data presented in this study are available on a reasonable request from the corresponding author.
